# Connecting working and long-term memory: Bayesian-hierarchical multinomial model-based analyses reveal storage next to retrieval differences

**DOI:** 10.3758/s13421-024-01627-3

**Published:** 2024-09-05

**Authors:** Carolin Streitberger, Beatrice G. Kuhlmann, Matt E. Meier, Nina R. Arnold

**Affiliations:** 1 Department of Psychology, University of Mannheim, Mannheim, Germany; 2 Department of Psychology, Appalachian State University, Boone, NC USA; 3 Medical Faculty Mannheim/Heidelberg University, Central Institute of Mental Health, Mannheim, Germany

**Keywords:** Working memory capacity, Episodic long-term memory, Associative memory, Storage-retrieval model, Multinomial processing tree model

## Abstract

Individual differences in working memory capacity (WMC) are correlated with long-term memory (LTM) differences. Whether this is because high-WMC individuals encode more effectively, resulting in better LTM storage, or because they better retrieve information from LTM is debated. In two experiments, we used Bayesian-hierarchical multinomial modeling to correlate participant-level storage and retrieval processes from LTM recall to WMC abilities estimated from operation and symmetry complex span tasks. In Experiment 1, we presented participants with 20 consecutive words, including semantically associated pairs (e.g., *knife* and *fork*), to assess LTM processes. Participants received standard (*n* = 242) or associative-storage instructions (*n* = 222) and then completed a free recall task. In Experiment 2, we instructed participants (*N* = 239) to memorize 40 cue-target words as pairs before completing free and cued recall tasks. Correlations with WMC emerged with storage and retrieval processes and only when an associative storage strategy was instructed (Experiment 1). When associative processing was inherent to the task (Experiment 2), only the associative storage, not the retrieval advantage, replicated. The strategy reports suggest that high-WMC individuals use associative encoding strategies more effectively, resulting in better storage in LTM.

**Supplementary Information:**

The online version contains supplementary material available at 10.3758/s13421-024-01627-3.

## Introduction

When we want to remember information, like a phone number, we actively rehearse it to keep it in our limited working memory store because we otherwise forget it quickly. However, storage in long-term memory (LTM) is unlimited and permanent (e.g., Atkinson & Shiffrin, 1968; Baddeley & Hitch, 1974). Correlational and latent-variable individual-differences approaches consistently show that higher working memory capacity (WMC) relates to better performance on LTM tasks. For example, studies find this WMC-LTM connection to hold independent of the specific recall task, complex span task, associated or not associated word material, and correlational or quartile-split analyses (Bailey et al., 2008; Brewer & Unsworth, 2012; Spillers & Unsworth, 2011; Unsworth, 2007, 2009a, 2009c; Unsworth et al., 2011, 2014, 2015; Unsworth & Brewer, 2009; Unsworth & Spillers, 2010b).

Past research on the connection between WMC and LTM has primarily used performance-based LTM measures that rely on multiple cognitive processes. To better explain the observed correlations, it is essential to distinguish between the storage processes that occur during study and the retrieval processes that occur during recall. Without distinguishing between them, storage and retrieval processes may cancel each other out, such that there are null effects on a behavioral level, which can lead to misinterpretations (Batchelder & Riefer, 1980). Therefore, it is important to determine whether the correlations between LTM and WMC performance are due to differences in underlying storage and/or retrieval processes.

These correlations may be due to a retrieval advantage, as some have suggested for the association between intelligence and memory (Mogle et al., 2008). A prominent theoretical account of the WMC-related retrieval advantage is controlled cue use: for episodic memory, high-WMC individuals strategically use contextual and temporal cues generated while studying during recall (Unsworth & Engle, 2006, 2007b). And for semantic memory, they use cues hierarchically to search groups of clusters and then their objects (Rosen & Engle, 1997; Unsworth & Engle, 2007a).

In contrast, low-WMC individuals retrieve items more automatically without effectively using cues to reduce the search sets. Therefore their search sets include irrelevant items, leading to worse episodic recall performance in terms of proportion recalled, speed, and number of intrusions (also evidenced by a simulation study; Unsworth, 2007; Unsworth & Engle, 2007b). Unlike in free recall tasks described above, cued recall requires less use of contextual and temporal cues for retrieval due to the presence of an external cue, providing environmental support (Craik, 1983). Therefore, finding that WMC is less strongly related to cued than free recall (i.e., a test that depends relatively little on retrieval vs. a test that depends more on retrieval; Unsworth, 2009a) also suggests that a retrieval advantage underlies the WMC-LTM correlation.

However, other studies provide evidence for an encoding advantage of high-WMC individuals, resulting in better LTM storage. High-WMC individuals better attend to relevant information at encoding, especially during distraction (Engle & Kane, 2004; Unsworth et al., 2010). High-WMC individuals also use more effective encoding strategies, such as mental imagery or generating sentences, which contributes to the WMC-LTM relationship (Bailey et al., 2008; Unsworth & Spillers, 2010a). Yet, some research has found evidence supporting both WMC-related storage and retrieval advantages: high-WMC individuals employ distinct encoding strategies, select a more constrained search set, and exhibit better monitoring of their output during retrieval (Unsworth, 2016).

Although comparing the WMC-LTM correlation in free versus cued recall (Unsworth, 2009b) and under different encoding instructions (e.g., Unsworth & Spillers, 2010a) provides insights into the extent to which WMC is related to LTM retrieval versus encoding processes, this evidence is indirect. An approach providing direct and separate measures of LTM storage and retrieval processes is via cognitive modeling. Multinomial processing tree (MPT) models have been developed for this purpose (Batchelder & Riefer, 1980; Riefer & Rouder, 1992; Rouder & Batchelder, 1998). For example, the free-then-cued recall MPT model (Riefer & Rouder, 1992; Rouder & Batchelder, 1998) follows the logic that cued recall makes fewer demands on self-generated retrieval cues than free recall. Thus, comparing free versus cued recall performance allows inferences about retrieval processes. Crucially, MPT models make these assumptions about the involved storage and retrieval processes by explicitly stating processing paths through which these parameters connect to the observable responses on LTM memory tests. This allows for parameter estimation from observed free and cued recall data. In the current study, we applied two different LTM paradigms and associated MPT models that enable the estimation of storage and retrieval processes and relate these estimates to WMC. We describe these models and the associated LTM paradigms in more detail in the Experiment sections.

In the only previous study applying an MPT model to connect WMC and LTM storage and retrieval processes, WMC was related only to the model’s storage but not its retrieval parameters (Marevic et al., 2018). However, this study implemented an intentional forgetting paradigm and instructed participants to remember or forget the item directly after seeing each item. Thus, the observed correlation with LTM storage may specifically reflect the high-WMC individuals’ better attention control in this specific setting (e.g., Colflesh & Conway, 2007; Kane et al., 2001; Kane & Engle, 2003). Further, WMC ability was assessed with two verbal span tasks, meaning that the storage advantage may be confounded as both the LTM and the WMC tasks used verbal materials. Thus, the MPT model-based approach seems promising for better understanding the WMC-LTM connection, and the specific relation to MPT-derived parameters calls for replication and more thorough investigation across different LTM paradigms.

In the current experiments, we investigated whether WMC correlates with the MPT-derived LTM storage and/or retrieval process estimates. We used verbal and visuospatial WMC tasks and different LTM tasks to test the robustness of this relation.

## Experiment 1

**Fig. 1:**
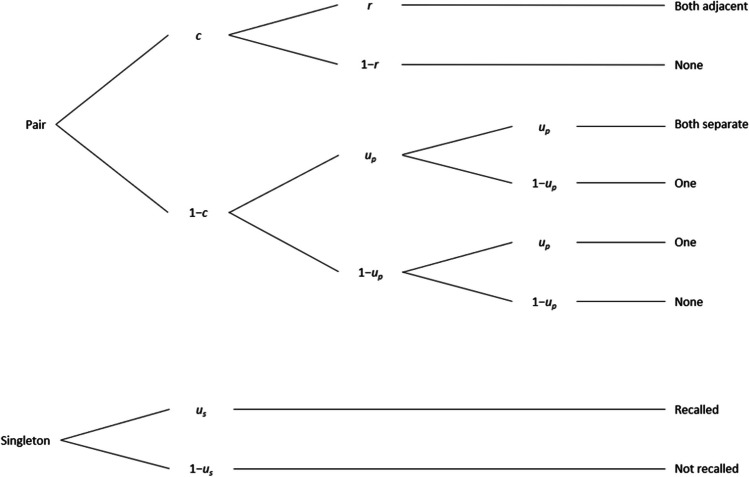
Experiment 1: Pair-clustering multinomial processing tree model for free recall of word lists including semantically related word pairs and singletons, originally proposed by Batchelder and Riefer (1980). *c* = probability of storing semantically related words together in a cluster; *r* = probability of retrieving stored word clusters at test; *u*_*p*_ = probability of storing and retrieving individual words of a semantically related pair that was not clustered; *u*_*s*_ = probability of storing and retrieving singleton words (i.e., no semantically related partner word on the list)

Experiment 1 applied the first multinomial model developed to separate storage versus retrieval processes in LTM: the pair-clustering model by Batchelder and Riefer (1980). This model, depicted in Fig. 1, can be applied to free recall data from one study-test trial. The study list contains several unique pairs of semantically associated words (e.g., *knife* and *fork*); however, each word is presented individually for study with lags between the associated words. The list also contains so-called singleton words not associated with any other study word. Of most interest is recall performance for words from the semantically associated pairs: Participants may recall (a) both pair words adjacently, (b) both words separated, (c) only one word, or (d) neither word. As illustrated in the first tree in Fig. 1, the model estimates the probability of storing semantically associated words together as one cluster in LTM (parameter *c*) and the probability of retrieving such stored clusters from LTM at test (parameter *r*) from these recall-event frequencies. In the first branch, the sequence of successful storage of two semantically associated words as a cluster and subsequent successful retrieval (i.e., c*r) results in adjacent recall of both words.

In contrast, if only cluster storage but not its retrieval is successful (i.e., c*[1−*r*]), both words are *not* recalled. If the two words are not stored as a cluster, with probability (1−*c*), parameter *u*_*p*_ captures the probability of both successfully storing and retrieving each individual pair word, resulting in either successful yet separate recall of both words, recall of only one of the words, or recall of neither word. For singleton words (second tree in Fig. 1), parameter *u*_*s*_ similarly captures the probability of both storage and retrieval. Central to our research question are parameters *c* and *r*, measuring the probability of associative storage versus retrieval, respectively. The *u* parameters are hybrid and do not separate storage from retrieval processes. Thus, they are not of particular interest to us but are needed for accurate modeling of *c* and *r*.

Note that *c* and *r* measure processes specific to storing and retrieving clustered information in associative memory. The parameter *c* specifically measures associative storage processes involved in inter-item relational processing (Hunt & Einstein, 1981) but not item-specific elaboration of single words (which would affect *u*). Without further instructions, parameter *c* may be influenced by individual differences in associative encoding strategy production. That is, *c* may be low not due to poor associative encoding, but rather because of non-relational encoding despite good item-specific elaboration. To account for these potential discrepancies in encoding strategies, we explicitly instructed some participants to engage in inter-item relational processing during encoding. Further, we included a nonverbal complex span task, the symmetry span, to minimize strategy overlap and method variance more generally between the WMC and LTM tasks.

While *c* measures the probability of storage of clusters, *r* measures the probability of retrieval of these clusters formed through relational processing during study. The WMC-related difference in retrieval was proposed specifically for semantically associated clusters (Unsworth et al., 2013), representing the retrieval process that the *r* parameter taps into.

### Method

#### Participants and design

Out of 485 undergraduates participating for course credit at the University of North Carolina at Greensboro (UNCG) and Western Carolina University (WCU), 464 were included in our analyses because they had at least one valid span score. Additional inclusion criteria were age between 18 and 35 years, English as a native language, and no visual impairments. Instructions were manipulated between participants with random assignment: 242 received standard memory instructions, whereas 222 were instructed to attend to semantically associated word pairs during study. Both conditions had similar proportions of participants from UNCG (40.91% in the standard condition; 39.19% in the strategy condition) and WCU (59.09% in the standard condition; 60.81% in the strategy condition), χ^2^(1, *N* = 464) = .08, *p* = .78.

#### Material and procedure

The procedure differed slightly between UNCG and WCU. At UNCG, participants volunteered to participate in a two-session study, each lasting less than 1.5 hr. At WCU, participants volunteered to complete one 2-hr session. At both institutions, we administered the operation span first, the symmetry span third, and the recall task last. These tasks were identical across testing sites. The second task was an unrelated task for other projects.

##### Complex span tasks

WMC was assessed with the automated operation and symmetry span tasks (Unsworth et al., 2005). In these tasks, participants were asked to memorize lists of items while completing interleaved processing tasks. Then, they were asked to recall the items in the correct serial order of presentation. During the preceding practice phase, participants completed the memorization and distractor tasks separately and interleaved. From the distractor-only practice, an individualized response deadline was computed. The response deadline was used during the scored portion of the task: if a participant exceeded this response deadline, the trial was scored as an error. Participants were instructed to get more than 85% of the distractor trials correct for us to use their data (Conway et al., 2005).

The operation span was split into three blocks, each including five lists (list lengths three to seven), where the to-be-memorized-items were letters, and the distractor tasks were arithmetic problems. Similarly, the symmetry span also had three blocks but with four lists (list lengths two to five), where participants remembered the location of red squares in a grid and the distractor task was vertical symmetry judgments of shapes in a similar grid. Participants did not know the length of the lists at the onset of a trial.

##### Recall task

We assessed LTM with a free recall task. Before the study phase, we instructed participants to carefully study the upcoming word list for a memory test. Participants in the associative strategy condition were informed that some words are semantically associated (e.g., *knife* and *fork*) and that it would be helpful to remember these in pairs. Then, all participants studied the same list: 20 words were presented one at a time in a black font on the center of a white screen for 3 s, followed by a 500-ms blank screen. The 20 words included 16 pair words (i.e., eight pairs) and four singletons. The words were drawn from category norms (Van Overschelde et al., 2004). For the semantically associated pairs, two words (three to six letters, *M* = 4.75, *SD* = 1.75) each were selected from the top three positions of eight categories (59–96% naming rate, *M* = 79.00a, *SD* = 12.21). For the singletons, one word each was selected from these ranks from four additional categories to match the pair words in length (three to six letters, *M* = 4.75, *SD* = 1.75) and category typicality (55–99% naming rate, *M* = 79.00, *SD* = 12.21). The word order was randomized by participants with the restriction that words from a semantically associated pair were spaced by at least one to at most four intervening other-category words. After the study phase, participants worked on a self-paced filler task for 1 min, where they had to count backwards by typing in the result from a three-digit number in steps of three. In the ensuing recall test, participants were asked to type all words they could remember from the study list, in whatever order they came to mind, for 2 min. Previously typed words were displayed on the screen. Then, participants were debriefed, compensated, and dismissed.

### Results and discussion

Mean operation (*M*_standard_ = 50.10, *SD*_standard_ = 14.95, *M*_strategy_ = 51.28, *SD*_strategy_ = 15.16) and symmetry span (*M*_standard_ = 26.93, *SD*_standard_ = 7.40, *M*_strategy_ = 27.48, *SD*_strategy_ = 7.84) performance were comparable to published norms (Redick et al., 2012) and did not significantly differ between the conditions, *t*_*Operation*_(404.52) = -0.79, *p* = .43 and *t*_*Symmetry*_(410.18) = -0.73, *p* = .46. For the subsample with valid scores on both span tasks, operation and symmetry span scores were correlated, *r*(364) = .39, *p* < .001. Thus, we *z*-standardized and averaged participants’ operation and symmetry span scores for a WMC composite score. For the remaining participants who only had one of two valid span scores (21.90% in the standard condition; 20.27% in the strategy condition), we used the *z*-transform of that score as a proxy.

Reliability estimates of the span tasks were acceptable. Cronbach’s alpha estimates were α = .84 for the operation and α = .69 for the symmetry span and previous studies found values between α = .78 and α = .87 (Foster et al., 2015; Unsworth et al., 2005).

#### Recall performance

To code the raw data, we used the R package stringdist (R Core Team, 2020; Van der Loo, 2014), allowing verbatim recall with one typo. Table 1 presents the means and confidence intervals of the proportion recalled and the Adjusted Ratio of Clustering (ARC; Roenker et al., 1971). The ARC describes to what extent the recall output contains category repetitions (i.e., pair clusters) relative to chance expectancy (range -1 to +1 with 0 indicating chance clustering). The strategy condition’s significantly higher ARC scores, *t*(350.23) = -2.95, *p* = .003 show that they followed the instructions and indeed clustered more. However, this advantage did not translate into a memory performance benefit in terms of proportion recalled: participants who got strategy instructions did not recall more words than participants who got standard instructions, *t*(460.92) = -1.25, *p* = .21.

Of central interest were correlations with WMC, also presented in Table 1: proportion recalled correlated significantly with WMC in both conditions, *r*_*standard*_(240) = .18, *p* = .01 and *r*_*strategy*_(220) = .17, *p* = .01, replicating previous research. ARC scores correlated with WMC only in the strategy condition, not in the standard condition, *r*_*standard*_(207) = .05, *p* = .48 and *r*_*strategy*_(190) = .19, *p* = .01.

**Table 1:** Means and correlations of performance measures and parameter estimates for Experiment 1

	Mean			WMC correlation	
	Standard	Strategy	Difference	Standard	Strategy
Proportion recall	.53 [.51, .55]	.55 [.53, .57]	.02 [-.05, .01]	.18 [.05, .30]	.17 [.04, .29]
ARC	.15 [.02, .28]	.51 [.33, .70]	.36 [.12, .60]	.05 [-.09, .18]	.19 [.05, .33]
*c*	.32 [.26, .37]	.41 [.34, .46]	.09 [.00, .17]	.02 [-.11, .14]	.13 [.03, .23]
*r*	.50 [.41, .63]	.59 [.50, .70]	.08 [-.07, .23]	.07 [-.04, .18]	.12 [.01, .23]
*u*_*p*_	.52 [.48, .56]	.51 [.46, .55]	-.02 [-.07, .04]	.07 [-.05, .19]	.07 [-.11, .21]
*u*_*s*_	.63 [.59, .66]	.57 [.53, .61]	-.05 [-.10, .00]	.07 [-.04, .18]	-.04 [-.17, .09]

*Note.* "Standard" is the standard memory condition and "Strategy" is the associative strategy storage condition. "Difference" refers to the mean absolute difference of the two conditions. "ARC" is the Adjusted Ratio of Clustering (Roenker et al., 1971). Square brackets indicate 95% confidence intervals for performance measures (proportion recall and ARC) and 95% Bayesian credibility intervals for model-based parameters (*c, r, u*_*p*_*, u*_*s*_). Correlations between WMC and performance measures are based on Pearson’s *r*. Model parameters and their correlations were estimated with the Bayesian-hierarchical latent-trait approach for MPT (Klauer, 2010) in TreeBUGS (Heck et al., 2018). ARC means and correlations could not be computed for *n*_*standard*_ = 29 and *n*_*strategy*_ = 24. All other reported values are based on *n*_*standard*_ = 242 and *n*_*strategy*_ = 222

#### Model-based analysis

To disentangle the underlying processes storage from retrieval, we used the latent-trait MPT approach from the TreeBUGS package in R (Heck et al., 2018; Klauer, 2010; R Core Team, 2020) to derive individual and group-mean estimates of the pair-clustering model parameters in each instruction condition as well as to estimate the correlations of these parameter estimates with the span scores. TreeBUGS uses JAGS's basic Markov chain Monte Carlo (MCMC) algorithm to draw posterior distribution samples (Plummer, 2003). We started with 20,000 samples, with 2,000 samples as burn-in and adaptation samples, and retained every fifth sample. We then iteratively added 10,000 samples with 1,000 adaptation samples until all estimates converged (i.e., $\widehat{R}$ < 1.05 and effective samples > 2000). The T1 and T2 statistics (Klauer, 2010) indicated a good model fit, all *p* > .05. Regarding the correlations, TreeBUGS repeatedly computes the correlations for all posteriors on the latent probit scale. To test parameter differences and correlations, we used Bayesian *p* values, also calculated by TreeBUGS. A Bayesian *p* summarizes the posterior distribution as it represents how much of the posterior is less than zero, i.e., how much of the proportion of samples from the posterior is not in line with the null hypothesis, and can therefore be compared to the chosen significance level (Heck et al., 2018). Furthermore, to summarize the posterior distributions of the effects, the 95% Bayesian credibility intervals are displayed alongside the mean estimate for each model parameter in Tables 1 and 2.

**Table 2:** Means and correlations of performance measures and parameter estimates for Experiment 2

	Mean	WMC correlation
Proportion free recall	.32 [.31, .33]	.15 [.02, .27]
Proportion cued recall	.60 [.58, .63]	.13 [.01, .26]
*a*	.63 [.60, .66]	.11 [.04, .17]
*r*	.49 [.47, .51]	-.07 [-.17, .04]
*s*	.03 [.02, .04]	-.07 [-.16, .03]
*u*	.03 [.03, .04]	-.04 [-.16, .10]
*f*	.02 [.01, .03]	-.08 [-.19, .06]

*Note.* Square brackets indicate 95% confidence intervals for performance measures (proportion recall) and 95% Bayesian credibility intervals for model-based parameters (*a, r, s, u, f*). Correlations between WMC and performance measures are based on Pearson’s *r*. Model parameters and their correlations were estimated with the Bayesian-hierarchical latent-trait approach for MPT (Klauer, 2010) in TreeBUGS (Heck et al., 2018). All estimates are based on *N* = 239

Mean parameter estimates and their correlations with WMC are also shown in Table 1. For group comparisons, positive values again indicate higher values in the instructed condition. We can conclude that the associative encoding strategy increased the probability of pair storage (parameter *c*) by .09 compared to participants in the standard condition, Bayesian *p* = .02. Additionally, there was a significant positive correlation of the pair storage parameter *c* with WMC in the strategy condition, Bayesian* p* = .005 but not in the standard condition, Bayesian *p* = .39. That is, only in the strategy condition, participants with higher WMC showed increased cluster storage probability compared to participants with lower WMC. The manipulation did not affect retrieval (parameter *r,* Bayesian *p* = .12), and again, there was a significant correlation with WMC in the strategy condition only, *r* = .12, Bayesian *p* = .02, and not in the standard condition, Bayesian *p* = .11. This indicates that although both groups retrieved with the same probability, only high-WMC participants in the strategy condition showed a retrieval advantage compared to low-WMC participants. For individual word-memory processes given unsuccessful clustering (parameter *u*_*p*_), there was no difference between conditions, Bayesian *p* = .29, and no correlations to WMC in the standard condition, Bayesian *p* = .12, nor in the strategy condition, Bayesian *p* = .18. For singletons, the joint storage and retrieval probability (parameter *u*_*s*_) is higher in the standard condition, Bayesian *p* = .02, but does not correlate with WMC in the standard condition, Bayesian *p* = .10, nor in the strategy condition, Bayesian *p* = .74.

The results of Experiment 1 suggest a relation between WMC and associative storage and retrieval, but only when participants were instructed to cluster via strategy instructions.

In Experiment 2, we queried participants to report their spontaneous strategy use to better understand if the associative storage or retrieval advantages stem from qualitatively different strategy use or indeed reflect better storage and retrieval when using the same strategies as the current results suggest. We used weakly associated word pairs, and because associated word material leads to associative encoding, it is possible to replicate the WMC-related associative storage and retrieval advantages without explicit strategy instructions.

## Experiment 2

For Experiment 2, we used a model to separate storage from retrieval processes from free-then-cued recall of cue-target word pairs, developed and validated by Rouder and Batchelder (1998; see also Riefer & Rouder, 1992). In this paradigm, participants study a list of word pairs (both cues and target words presented simultaneously next to each other during study) and then try to freely recall all words before being cued with the cue word of each pair. Observable data categories are all possible combinations of free recall of both, just one, or no word from a pair with the successful or failed cued recall of this pair.

**Fig. 2:**
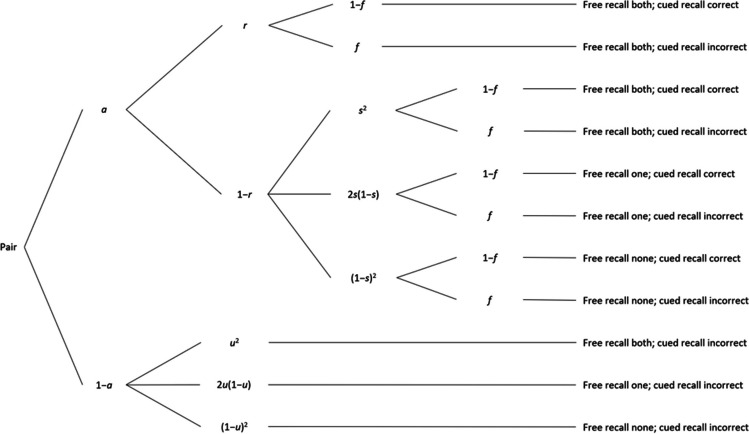
Experiment 2: Multinomial processing tree model of storage and retrieval processes in free-then-cued recall of a list of word pairs, originally proposed by Rouder and Batchelder (1998). *a* = probability of associative storage of pair; *r* = probability of associative retrieval during free recall; *s* = probability of single-word retrieval during free recall; *u* = probability of single-word storage and retrieval during free recall; *f* = forgetting the pair association due to the time delay between free and cued recall

Based on these frequencies, the model, depicted in Fig. 2, estimates the probability of associative storage of a cue-target pair (parameter *a*) and the probability of successfully retrieving a stored cue-target pair in free recall (parameter *r*), comparable to the *c* and *r* parameters of the pair-clustering model in Experiment 1. Other processes needed to explain the data are freely retrieving single words after they were stored but not retrieved as a pair (parameter *s*), freely retrieving single-words (i.e., words that were not stored as a pair originally; hybrid parameter *u*, comparable to *u*_*p*_ in Experiment 1), and possible forgetting of the cue-target association due to the time delay between the free and the cued test (parameter *f*). In the first branch of the model in Fig. 2, both associative storage and retrieval lead to free recall of both pair words and successful cued recall. If a pair is associatively stored into LTM, cued recall is successful unless the stored relation is forgotten until the cued recall with probability *f*. However, even if free retrieval of the pair fails, both (probability s*s) or just one (probability s*[1−*s*]) of the words may still be retrieved individually. Additionally, retrieving neither word is possible (probability [1−*s*]*[1−*s*]). If a pair is not associatively stored, cued recall will fail, but successful single-word storage and free retrieval may nonetheless lead to free recall of both pair words (probability u*u) or just one (probability u*[1−*u*]), or may fail for both single words (probability [1−*u*]*[1−*u*]), in which case both recall attempts fail for this pair. Because both pair words are presented next to each other and are to be studied as a pair, all participants should intentionally engage in associative storage as in the strategy condition in Experiment 1. Thus, we expected to replicate the positive correlation between WMC and associative storage (parameter *a*) but not retrieval (parameter *r*).

### Method

#### Participants and design

A total of 249 undergraduates at WCU participated for course credit, and in the analyses we included 239 who had at least one valid span score. Further inclusion criteria were age between 18 and 35 years, English as a native language, and no visual impairments. All participants first received a free recall test followed by a cued recall test. There were no between-subject manipulations.

#### Material and procedure

Participants completed a 1-hr session, where they first did the symmetry span, then the recall task, and lastly the operation span.

##### Complex span tasks

We used the same tasks to assess WMC as in Experiment 1, but due to a programming error that affected the scoring and feedback after the first block in the task version ran for this experiment, some participants received inaccurate feedback on their performance for the second and third blocks. Although this erroneous feedback probably did not impact span performance in these blocks, we made the conservative decision to only use data from the first block. We deem this unproblematic because using the first blocks of the operation and symmetry span explains 78.5% of the variance in fluid intelligence accounted for by a battery of the three-blocked operation, symmetry, and rotation span tasks, and has thus been suggested as a reliable shortened WMC assessment (Foster et al., 2015).

To assess reliability, we again calculated Cronbach’s alpha and found α = .63 for the shortened operation and α = .38 for the shortened symmetry span, whereas previous studies using the shortened tasks found values between α = .52 and α = .72 (Beikmohamadi & Meier, 2024; Goller et al., 2020). Therefore, we regard the operation span as reliable, but the symmetry span is unsatisfactory. To ensure that the correlations with the storage and retrieval parameters are not affected by the low reliability, we re-ran the model with just the *z*-transformed operation span task performance as the covariate and found the same results pattern (see Online Supplemental Material). Therefore, we have confidence in our adjusted (shortened) measure of WMC.

##### Recall task

Unlike in Experiment 1, the instructions for the free-then-cued-recall task emphasized the need to study the word pairs as a whole. Both words of each pair were presented together (i.e., cue word-target word) for 6 s in black font on the center of a white screen, followed by a blank screen for 500 ms. Because there was no experimental manipulation, all participants learned the same 20 weakly associated word pairs (Carpenter et al., 2006). Words were between four and seven letters long (*M* = 5.68, *SD* = 0.76) with a forward and backward association strength between .01 and .06 according to the USF word-association norms (Nelson et al., 2004; forward: *M* = .03, *SD* = .02; backward: *M* = .03, *SD* = .01). After presentation of the last pair, participants completed the same filler task as in Experiment 1, in which they counted backwards in steps of three from a provided three-digit number for 1 min. Then, participants completed two recall tests: first, a free recall test in which they typed words in any order that they could remember for 3 min. Next, for the cued recall test, the 20 cue words appeared in boxes on the screen in random order, where participants could enter the corresponding target words for 3 min. Already entered target words remained on the screen next to the cue.

Finally, participants were asked to indicate their frequency of using (from 1 = *no use* to 7 = *extensive use*) 11 possible encoding strategies (e.g., making associations between both cue and target, or forming sentences with just the cue or the target word, etc.), based on an encoding-strategy questionnaire developed for word pairs (Finley & Benjamin, 2012). They were then debriefed, compensated, and dismissed.

### Results and discussion

Span task performance (operation span:* M* = 16.45, *SD* = 5.47; symmetry span: *M* = 7.86, *SD* = 2.90) here cannot be compared to published norms because we are using data from one block only. However, operation and symmetry span scores were correlated, *r*(234) = .41, *p* < .001, and performance is comparable to studies that also only used one block of the operation and symmetry span (Beikmohamadi & Meier, 2024; Goller et al., 2020). We again computed the WMC composite based on the *z*-standardized and averaged scores for participants with valid scores on both tasks. For participants with only one valid span score (1.26%), we again used their *z*-transform.

#### Recall performance

Just like in Experiment 1, for preprocessing of the raw data, we used the R package stringdist (R Core Team, 2020; Van der Loo, 2014) and allowed verbatim recall with one typo. Table 2 shows the means and 95% confidence intervals for the proportion recalled for free recall and cued recall, as well as their correlations with WMC. The correlations are similar to Experiment 1, *r*_*free*_(237) = .15, *p* = .02 and *r*_*cued*_(237) = .13, *p* = .04. Next, we examined the associations with the storage and retrieval parameters, of which the proportion recalled is a conglomerate and thus a less sensitive measure.

#### Model-based analysis

As described for Experiment 1, we used the same MCMC-based parameter estimation procedure in TreeBUGS with the latent-trait MPT function (Heck et al., 2018; Klauer, 2010; R Core Team, 2020) until the model converged ($\widehat{R}$ < 1.05, and T1 and T2 *p* > .05). Table 2 also presents the mean parameter estimates and the estimated correlations with WMC and the corresponding 95% Bayesian credibility intervals. Similar to Experiment 1, we observed a positive correlation between storage (parameter *a*) and WMC, Bayesian *p* = .001: high-WMC individuals had a higher probability of storing the cue-target association than low-WMC individuals. There was no evidence for a WMC-related retrieval advantage (neither in retrieving stored pairs, parameter *r*, Bayesian *p* = .91, nor in retrieving individual words, parameter *s,* Bayesian *p* = .92) and also no advantage in forgetting (*f,* Bayesian *p* = .89) or singleton memory (*u,* Bayesian *p* = .76). Thus, the current results replicate the WMC advantage in associative storage in LTM observed in Experiment 1. Notably, they do so without having instructed a specific associative encoding strategy.

#### Strategy reports

The observed WMC-related advantage in associative storage may reflect a qualitative difference in encoding strategy use with high-WMC individuals more frequently using associative strategies and/or a quantitative difference such that storage is more effective even when using the same strategies. To address this, we queried participants about their encoding strategy use at the end of the experiment. Use frequency significantly differed across the 11 encoding strategies, *F*(10, 2618) = 127.80, *p* < .001. The least often used encoding strategy was making a sentence (*M* = 2.00, *SD* = 1.57), whereas the most often used strategy was making an association between cue and target (*M* = 5.97, *SD* = 1.54). Table 3 shows the frequency means and correlations with WMC. To summarize, all correlations were between *r* = -.13 and *r* = .05 and all *p* ≥ .05, suggesting that, although participants use some encoding strategies more than others, this was unrelated to WMC.

**Table 3:** Means and correlations of encoding strategies for Experiment 2

Strategy type	Strategy	Mean	WMC correlation
Associative	Cue-Target Association	5.97 [5.77, 6.17]	.05 [-.08, .18]]
	Interactive Imagery	4.49 [4.22, 4.75]	-.13 [-.25, .00]
	Interactive Sentence	2.44 [2.19, 2.70]	-.06 [-.19, .06]
Non-associative	Single Focus	3.74 [3.50, 3.99]	.00 [-.13, .13]
	Mental Imagery	3.02 [2.77, 3.27]	-.08 [-.20, .05]
	Sentence	2.00 [1.80, 2.20]	-.09 [-.22, .03]
Not specified	Rote Rehearsal	5.69 [5.46, 5.92]	.03 [-.10, .15]
	Observation	3.53 [3.29, 3.77]	.00 [-.13, .13]
	Personal Significance	2.90 [2.67, 3.14]	-.11 [-.23, .02]
	Between-Pair Association	2.77 [2.52, 3.01]	-.02 [-.15, .11]
	Story	2.10 [1.86, 2.33]	.00 [-.13, .12]

*Note.* "Associative" strategies focused on encoding the association between cue and target and "Non-associative" strategies focused on encoding either the cue or the target. "Not specified" are strategies that cannot be categorized into associative or non-associative. Square brackets indicate 95% confidence intervals. Correlations with WMC are based on Spearman’s ρ. All estimates are based on *N* = 239

In Table 3, we classified these encoding strategies by whether they clearly have an associative nature (e.g., the cue-target association strategy is "made associations between the left-hand word and right-hand word in a pair"), or are non-associative (e.g., the sentence strategy is "formed a sentence with individual words but not with both words in a pair together"), or are non-specifiable strategies (e.g., the between-pair association strategy is "made associations between the words across multiple pairs"). The associative strategies were used most often (*M* = 4.30, *SD* = 2.38), followed by non-specified strategies (*M* = 3.40, *SD* = 2.23), and then by non-associative strategies (*M* = 2.92, *SD* = 1.95). Again, there was a significant difference in use frequency across these three encoding strategies, *F*(2, 2626) = 73.71, *p* < .001, and no statistically significant correlations with WMC: all three correlations were between *r* = -.05 and *r* = -.02 and all *p* > .05. This is further evidence that both high- and low-WMC individuals used associative encoding strategies more than other strategies and to a similar degree.

Therefore, Experiment 2 is comparable to the condition instructing semantic associative encoding in Experiment 1, in which we also observed a WMC-related storage advantage. With the additional assessment of strategy use in Experiment 2, it is evident that this storage advantage is not a qualitative one related to high-WMC individuals using strategies for encoding associations more frequently. Rather, it is a quantitative advantage, such that when using similar (associative) strategies, high-WMC individuals store more effectively in LTM.

## General discussion

This research project aimed to connect LTM storage and retrieval processes to individual differences in WMC using two experimental paradigms and their corresponding MPT models. We applied Bayesian-hierarchical estimation methods to estimate individual MPT parameters and their correlations with WMC. We found a WMC-related storage advantage: significant correlations with WMC emerged for the storage parameters when associative storage was instructed (Experiment 1) or inherent to the task because cue-target word pairs were studied (Experiment 2). We also found a retrieval advantage for high-WMC individuals, but only in the pair-clustering paradigm when clustering was instructed (Experiment 1). Finding evidence for both WMC-related storage and retrieval effects is in line with previous work that finds WMC to affect multiple factors underlying storage and retrieval, such as using encoding strategies (corresponding to storage), efficiently selecting retrieval cues, and monitoring the recall output (corresponding to retrieval; Unsworth, 2016).

### Working memory capacity (WMC) and long-term memory (LTM) storage

In both experiments, we found evidence that WMC correlates with associative storage in LTM. This storage advantage may therefore explain the typical finding that high-WMC individuals perform better on LTM tasks than low-WMC individuals. Indeed, some researchers have suggested that WMC relates to LTM storage processes, specifically that differences in encoding strategy usage explain the relationship with LTM (Bailey et al., 2008; Unsworth & Spillers, 2010a). We found that associative storage is not higher because high-WMC individuals use different encoding strategies than low-WMC individuals: in Experiment 2, the frequency of using associative encoding strategies was not related to WMC, and everyone, independent of WMC, used associative encoding strategies more than non-associative ones. Further, in Experiment 1, the storage difference emerged when encoding strategy was instructed and was presumably similar across participants. Thus, it seems higher-WMC participants used the same associative encoding strategies as lower-WMC participants but used them more effectively, resulting in higher storage despite similar strategy use. This contrasts with previous findings (Bailey et al., 2008; Unsworth & Spillers, 2010a) reporting encoding strategy differences between high and low WMC participants when encoding single words. Thus, our observed similar encoding strategy use in high and low WMC participants in Experiment 2 may be specific to encoding word pairs for which using an associative strategy is obvious and thus was most frequent in all participants.

Consequently, the probability of associative storage was relatively high overall. Although in Experiment 1 the probability of storing a semantically associated pair was lower overall, semantic association was still likely an obvious strategy for this list, even without explicit instructions. We used short lags of one to four intervening other words between the two semantically associated words, which is known to facilitate pair storage (Batchelder & Riefer, 1980). Even under these optimal conditions, detecting and storing pairs presented separately as individual words depended on WMC when clustering was explicitly instructed (replicating Kuhlmann & Touron, 2016).

The present WMC-related storage effect may be specific to associative storage and depends on clustering instructions for individual words (Experiment 1) or the presentation of associated study material (word pairs in Experiment 2). However, the observed positive correlations between WMC and the storage parameters from two different MPT models provide clear evidence that LTM storage is related to WMC. Thus, theorizing about the WMC-LTM connection needs to consider such storage differences in addition to the focus on retrieval differences (Unsworth & Engle, 2007a). However, there are already studies considering storage-related aspects such as encoding strategies next to retrieval cues, finding that both storage and retrieval aspects are relevant (Bailey et al., 2008; Unsworth & Spillers, 2010a).

### WMC and LTM retrieval

In Experiment 1, we found that high-WMC individuals indeed retrieved stored information from LTM with a higher probability than low-WMC individuals. However, in Experiment 2, we find that WMC does not drive the retrieval advantage.

Prior work suggests high-WMC individuals efficiently select cues to reduce the number of irrelevant items to search through, increasing the probability of correct recall (Unsworth & Engle, 2007b). An error analysis of complex span data suggests that this efficiency is generated by selecting internally generated cues based on context and time to differentiate between different lists that they learned: items stored recently have a more similar context to the current retrieval phase and are therefore accessed (Unsworth & Engle, 2006). A WMC-related retrieval advantage has also been observed in recognition tests that provide external cues instead of solely relying on internal ones. When retrieval is controlled (recollection), a slower and more deliberate process, it loads on the WMC factor. However, when retrieval is fast and automatic (familiarity), there is no advantage related to WMC (Oberauer, 2005).

In the verbal fluency task, where participants must access semantic memory, individuals with high WMC have a retrieval advantage because they search for cues actively instead of passively (Rosen & Engle, 1997). Unsworth and Engle (2007a) showed in a re-analysis that the retrieval advantage in semantic memory may arise because high-WMC individuals use cues hierarchically to first access word clusters and then their items. They concluded that high-WMC individuals have better retrieval because they adapt cue use to the task's demands: using cues to reduce the items in episodic memory versus using cues to hierarchically guide the search in semantic memory.

Given this evidence for a WMC-related retrieval advantage, not finding this effect in Experiment 2 is somewhat surprising but could be due to underestimation of the correlation. To demonstrate that participants with higher WMC have increased storage but not retrieval probability, a direct test comparing the correlation coefficients across experiments would be needed. However, this would require more than 700 participants per group, based on the power analysis for a frequentist independent correlation test. Thus, we acknowledge that the correlation between WMC and LTM may be equally influenced by both underlying processes, but would like to highlight the crucial role of storage, which has been previously overlooked.

At the same time, the absent WMC-related retrieval effect is in line with an MPT analysis by Marevic et al. (2018). None of the studies cited above that argue for a WMC-related retrieval advantage derived separate process estimates of storage versus retrieval from a cognitive model. We encourage future studies employing cognitive modeling to investigate the relationship between WMC and retrieval from LTM. The inconsistent results in our experiments and in the literature imply that WMC-related retrieval effects may be non-general and that there may be boundary conditions. Future research should explore specific tasks settings and different models that may reveal a WMC-related retrieval advantage just like the pair-clustering (Batchelder & Riefer, 1980) but not the free-then-cued-recall model (Rouder & Batchelder, 1998) revealed this effect.

## Limitations

We used specific LTM tasks that may have fostered the observed WMC-related storage advantage. One potentially critical factor in the pair-clustering paradigm of Experiment 1 is the use of larger lags between the two semantically associated pair words than we used, as Batchelder and Riefer (1980) found better retrieval (but poorer storage) with larger lags. Under such conditions, a WMC-related retrieval advantage may emerge, unlike the short lags we used to foster associative storage. Likewise, in Experiment 2, the side-by-side presentation of the words as pairs resulted in the dominant use of associative encoding strategies. The observed WMC-related storage advantage may thus be specific to such conditions fostering associative processing at encoding.

Although we do not have support for a general robust WMC-related retrieval advantage, we do find that WMC is related to LTM retrieval in the pair-clustering paradigm under strategy instructions. We did not manipulate context features (e.g., background colors) in our experiments, which may increase the probability of WMC-related advantages in using contextual cues, as suggested by Unsworth and Spillers (2010a). So, it may be that the relation is visible under different study and test conditions or for different types of study material.

## Conclusion

We used Bayesian-hierarchical multinomial modeling to investigate the relationship between LTM storage and retrieval processes with WMC. We found that when intentionally processing associations, the probability of storage into episodic LTM is higher in high-WMC individuals than low-WMC individuals (in line with Bailey et al., 2008; Marevic et al., 2018; Unsworth & Spillers, 2010a, 2010b), but we did not find WMC-related differences in associative retrieval from episodic LTM (in contrast to Oberauer, 2005; Unsworth & Engle, 2006). The suggested WMC-related retrieval advantage may occur for shorter lists typically used in the research on WMC and LTM (but untypical for LTM research and not well suited for parameter estimation with MPT modeling) and/or under more difficult retrieval conditions (e.g., with interference built up across multiple lists) but the current results point to boundary conditions and thus are evidence against a general WMC-related retrieval advantage. The observed WMC-related storage advantage may be specific to associative storage but implies that storage should be considered in theorizing about the link between WMC and LTM.

## Supplementary Information

Below is the link to the electronic supplementary material.

Supplementary file1 (DOCX 39 KB)

## Data Availability

Data and materials are available in the OSF repository at https://osf.io/7dmxk/.

## Code availability

Code and analyses are available in the OSF repository at https://osf.io/7dmxk/.
