# Supplementary material for: Connecting working and long-term memory: Bayesian-hierarchical multinomial model-based analyses reveal storage next to retrieval differences
Source: Mem Cognit. 2024 Sep 5;52(8):1915–27. doi: 10.3758/s13421-024-01627-3 (PMC11588770; doi:10.3758/s13421-024-01627-3)
Supplement: Supplementary file 1 — Supplementary file1 (DOCX 39 KB) [file 13421_2024_1627_MOESM1_ESM.docx]

**Supplemental Material**

**Table A1**

*Means and correlations of parameters with just the operation span covariate from Experiment 2.*

|  | Mean | WMC correlation |
| --- | --- | --- |
| *a* | .63 [.60, .66] | .13 [.07, .20] |
| *r* | .49 [.47, .51] | -.09 [-.19, .02] |
| *s* | .03 [.02, .04] | -.10 [-.20, .00] |
| *u* | .03 [.02, .04] | -.03 [-.16, .10] |
| *f* | .02 [.01, .03] | -.09 [-.20, .04] |

*Note.* Square brackets indicate 95% Bayesian credibility. Model parameters and their correlations were estimated with the Bayesian-hierarchical latent-trait approach for MPT (Klauer, 2010) in TreeBUGS (Heck et al., 2018). All estimates are based on *N* = 237.
